# Metabolic Fingerprints from the Human Oral Microbiome Reveal a Vast Knowledge Gap of Secreted Small Peptidic Molecules

**DOI:** 10.1128/mSystems.00058-17

**Published:** 2017-07-18

**Authors:** Anna Edlund, Neha Garg, Hosein Mohimani, Alexey Gurevich, Xuesong He, Wenyuan Shi, Pieter C. Dorrestein, Jeffrey S. McLean

**Affiliations:** a Genomic Medicine Group, J. Craig Venter Institute, La Jolla, California, USA; b Collaborative Mass Spectrometry Innovation Center, Skaggs School of Pharmacy and Pharmaceutical Sciences, University of California, San Diego, La Jolla, California, USA; c Department of Computer Science and Engineering, University of California, San Diego, La Jolla, California, USA; d Center for Algorithmic Biotechnology, Institute of Translational Biomedicine, St. Petersburg State University, St. Petersburg, Russia; e School of Dentistry, University of California, Los Angeles, California, USA; f Department of Periodontics, University of Washington School of Dentistry, Seattle, Washington, USA; University of Trento

**Keywords:** *Lactobacillus*, *Streptococcus*, *Veillonella*, biofilms, oral microbiology, peptidic small molecules

## Abstract

Recent research indicates that the human microbiota play key roles in maintaining health by providing essential nutrients, providing immune education, and preventing pathogen expansion. Processes underlying the transition from a healthy human microbiome to a disease-associated microbiome are poorly understood, partially because of the potential influences from a wide diversity of bacterium-derived compounds that are illy defined. Here, we present the analysis of peptidic small molecules (SMs) secreted from bacteria and viewed from a temporal perspective. Through comparative analysis of mass spectral profiles from a collection of cultured oral isolates and an established *in vitro* multispecies oral community, we found that the production of SMs both delineates a temporal expression pattern and allows discrimination between bacterial isolates at the species level. Importantly, the majority of the identified molecules were of unknown identity, and only ~2.2% could be annotated and classified. The catalogue of bacterially produced SMs we obtained in this study reveals an undiscovered molecular world for which compound isolation and ecosystem testing will facilitate a better understanding of their roles in human health and disease.

**IMPORTANCE** Metabolomics is the ultimate tool for studies of microbial functions under any specific set of environmental conditions (D. S. Wishart, Nat Rev Drug Discov 45:473–484, 2016, https://doi.org/10.1038/nrd.2016.32). This is a great advance over studying genes alone, which only inform about metabolic potential. Approximately 25,000 compounds have been chemically characterized thus far; however, the richness of metabolites such as SMs has been estimated to be as high as 1 × 10^30^ in the biosphere (K. Garber, Nat Biotechnol 33:228–231, 2015, https://doi.org/10.1038/nbt.3161). Our classical, one-at-a-time activity-guided approach to compound identification continues to find the same known compounds and is also incredibly tedious, which represents a major bottleneck for global SM identification. These challenges have prompted new developments of databases and analysis tools that provide putative classifications of SMs by mass spectral alignments to already characterized tandem mass spectrometry spectra and databases containing structural information (e.g., PubChem and AntiMarin). In this study, we assessed secreted peptidic SMs (PSMs) from 27 oral bacterial isolates and a complex oral *in vitro* biofilm community of >100 species by using the Global Natural Products Social molecular Networking and the DEREPLICATOR infrastructures, which are methodologies that allow automated and putative annotation of PSMs. These approaches enabled the identification of an untapped resource of PSMs from oral bacteria showing species-unique patterns of secretion with putative matches to known bioactive compounds.

**Author Video**: An author video summary of this article is available.

## Abstract (precis)

Metabolomics is the ultimate tool for studies of microbial functions under any specific set of environmental conditions (D. S. Wishart, Nat Rev Drug Discov 45:473–484, 2016, https://doi.org/10.1038/nrd.2016.32). This is a great advance over studying genes alone, which only inform about metabolic potential. Approximately 25,000 compounds have been chemically characterized thus far; however, the richness of metabolites such as SMs has been estimated to be as high as 1 × 10^30^ in the biosphere (K. Garber, Nat Biotechnol 33:228–231, 2015, https://doi.org/10.1038/nbt.3161). Our classical, one-at-a-time activity-guided approach to compound identification continues to find the same known compounds and is also incredibly tedious, which represents a major bottleneck for global SM identification. These challenges have prompted new developments of databases and analysis tools that provide putative classifications of SMs by mass spectral alignments to already characterized tandem mass spectrometry spectra and databases containing structural information (e.g., PubChem and AntiMarin). In this study, we assessed secreted peptidic SMs (PSMs) from 27 oral bacterial isolates and a complex oral *in vitro* biofilm community of >100 species by using the Global Natural Products Social molecular Networking and the DEREPLICATOR infrastructures, which are methodologies that allow automated and putative annotation of PSMs. These approaches enabled the identification of an untapped resource of PSMs from oral bacteria showing species-unique patterns of secretion with putative matches to known bioactive compounds.

## INTRODUCTION

Host-microbiome interfaces are known to be of critical importance in structural, immunological, and metabolic functions (1, 64). These communication hot spots are key in human health and can be found in the oral cavity, which also provides the perfect portal of entry for microbes. Most of the approximately 1,000 oral bacterial species that have been identified so far are considered commensal. They have coevolved with their host and carry critical functions in training the immune system, protecting against epithelial cell injury, and suppressing pathogenic microbial growth (2). The last decade’s advances in sequencing technologies have revealed not only a heterogeneous distribution of bacterial taxa within the oral cavity (3, 4) but also between individuals within populations and between geographically distinct populations (e.g., the West versus Chinese) (5, 6). Each human mouth harbors a unique bacterial diversity consisting of on average approximately 150 bacterial taxa (5), and in addition to this complexity within each of these exclusive communities, all primary types of organism interaction can exist: i.e., consumer-resource interactions, competition, and mutualism (7, 8). Most of our knowledge of the human microbiome derives from associative large-scale DNA sequencing studies, which suggest that health-associated microbiomes are highly heterogeneous (65, 66) and that specific pathogens are associated with disease (9). Learning from previous studies, we now understand that microbiome research needs to move beyond correlation-based analysis, and we need to gain a deeper knowledge of molecular mechanisms supporting its complex network of functions. The social language (i.e., primary and secondary metabolites) of host-microbiome interactions needs to be identified, quantified, and ultimately functionally characterized. Numerous human microbiome-associated biosynthetic genes have been identified, which encode the major metabolic classes of SMs (10). These have a variety of biological functions, including antibacterial and immune modulation activities (10, 11). Antagonist activities between bacterial species associated with the human microbiome are mediated by SMs such as lantibiotics, bacteriocins, and microcins, which support both commensals and pathogens to compete and establish resilient colonization (10). The latter SMs are usually active against a narrow spectrum of Gram-positive bacteria that are closely related to the producing strain (10). Other SMs are host targets, such as *Escherichia coli*-produced enterotoxins (12) and modified amino acids (e.g., the neurotransmitter tryptamine) synthesized by gut bacteria (13). A few peptidic SMs (PSMs) that mediate antagonistic interactions between bacteria in the oral cavity have been isolated and structurally identified, such as mutanobactins (14), salivaricins (15), and proteases (17). However, the mechanisms that trigger the biosynthesis of these SMs in complex host-microbiome interactions *in vivo* are yet unknown. A few recent studies of human skin and gut reveal the potential of thousands of chemical species (10, 18). Here, we report on the temporal production and overall chemodiversity of the PSM secretome of human oral cavity-associated bacterial isolates belonging to the *Actinomyces*, *Fusobacterium*, *Lactobacillus*, *Porphyromonas*, *Streptococcus*, and *Veillonella* genera, as well as an *in vitro* oral biofilm model system containing more than 100 bacterial species (19, 20). Our previous comparative metagenomics and metatranscriptomics studies of the same biofilm model system confirmed its taxonomic and metabolic similarities to natural oral biofilms (19, 20). We also conducted parallel metatranscriptomics and global metabolomics (extracellular and internal) using gas chromatography-mass spectrometry (GC-MS) to study the interplay between gene expression and core metabolites. Here we expand on our knowledge of the *in vitro* biofilm community to include PSMs, which are well-known bioactive compounds that play key roles in cell-to-cell signaling and interactions with a host (21).

By employing liquid chromatography-tandem mass spectrometry (LC-MS/MS) on cell-free bacterial growth medium extracts, using the Global Natural Products Social Molecular Networking (GNPS) (22) and the DEREPLICATOR (23) infrastructures, we putatively identified a large number of PSMs secreted from the *in vitro* biofilm community as well as from individual bacterial isolates. We studied PSM secretion under different incubation conditions and incubation time points, including a rich SHI medium and a minimal chemically defined medium (cdm). The latter incubation medium supported an overall high metabolic activity and metabolite production but no growth in our previous study of the *in vitro* biofilm community (20). This condition is favorable for secondary metabolite production as most are produced during the stationary phase and not during exponential growth.

Together these results show a complex expression of SMs, whose role in human health and disease is yet unknown. The dynamic and species-specific production observed here implicates their potential roles in host-microbiome interactions and bacterial interspecies interactions. This work provides the first comprehensive landscape of unexplored PSMs produced by representatives of known commensal and pathogenic oral bacteria. The study lays the groundwork for further identification, classification, and investigation of novel groups of clinically and ecologically important PSMs.

## RESULTS AND DISCUSSION

**FIG 1:**
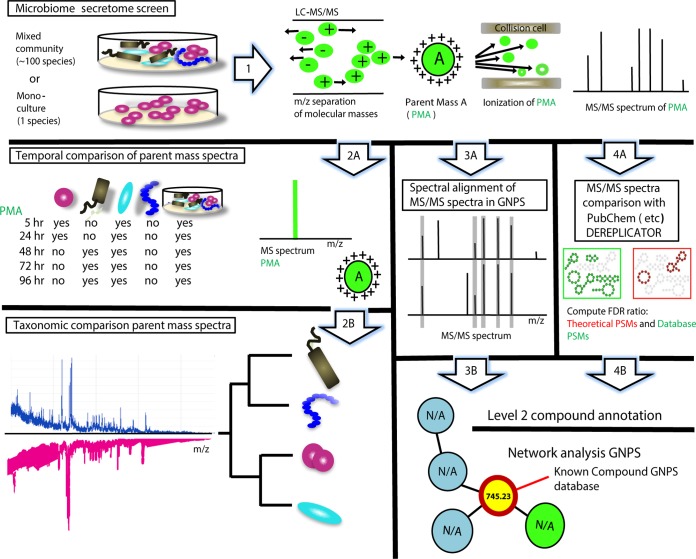
Flowchart of approaches employed to putatively annotate PMSs secreted from oral bacteria. (Step 1) A highly diverse *in vitro* biofilm community and single isolates of bacteria were grown in 1-ml cultures in a 24-well format. For the experimental setup, see Fig. S1. To identify parent masses and their corresponding ion fragment profiles in each sample, growth extracts were analyzed with an UltiMate 3000 UHPLC system and a Maxis qTOF mass spectrometer equipped with an electrospray ionization (ESI) source. (Steps 2A and B) Parent masses obtained from replicate samples were sorted into bucket tables and compared between time points (step 2A) and between bacterial isolates (step 2B) by using Venn diagrams and cluster analyses. (Steps 3A and B) The Global Natural Products Social Networks infrastructure (22) was employed to putatively annotate MS/MS spectra by spectral alignments of query spectra with ~20,000 benchmarked MS/MS spectra in the GNPS library (step 3A). GNPS networks revealed associations between query spectra and benchmark spectra, which contributed to level 2 annotations of ~50 PSMs (step 3B). (Steps 4A and B) The DEREPLICATOR tool (23) was used to annotate MS/MS spectra and predict the probability of each annotation by calculating false discovery rate (FDR) scores (step 4A). These annotations were based on structural homologies with PMS in databases such as PubMed and correspond to level 2 annotation standards (step 4B).

LC-MS/MS is a key analytical technology for detecting SMs of low molecular weight that cannot easily be structurally identified by genome sequencing (24, 25). Here we applied an ultrahigh-resolution–quadrupole time of flight mass spectrometry (UHR-qTOF MS) approach that yields high sensitivity regarding resolving closely spaced spectral peaks and detection of peaks with less intensity compared to standard methods. The approach allowed us to apply collision energy stepping coupled with the TOF transfer stepping, which facilitate thorough fragmentation of a diversity of molecules in a single LC-MS run. Given the relatively few studies comprehensively targeting identification and classification of secreted molecules of human oral bacterial species grown alone or existing as complex multispecies biofilm, our goal was to capture unique and previously unknown PSM signatures by applying the workflow presented in Fig. 1. We hypothesized that bacteria with distinct taxonomic backgrounds produce a rich diversity of PSMs and that some PSMs overlap other bacteria, while others are uniquely produced. This information can be used to target ecologically and clinically important PSMs and also to potentially taxonomically annotate SMs in the natural environment. We proposed that PSMs obtained from monocultures and mixed cultures of bacteria vary over time due to changes in metabolic activity across different stages of growth. Such a phenomenon is well acknowledged for all living organisms but largely underexplored for the human microbiome. In the first part of this study, we grew 27 bacterial isolates representing taxonomically broad groups of oral bacteria (e.g., *Streptococcus*, *Veillonella*, *Lactobacillus*, *Porphyromonas*, *Actinomyces*, and *Fusobacterium*) (see Fig. S1 in the supplemental material) and an already established *in vitro* oral biofilm model system (19, 20). This model system was previously shown to be both taxonomically and transcriptionally stable over time (19, 20), which allowed us to study PSM secretion on an hourly basis here. The study was conducted under anaerobic conditions in carbohydrate (i.e., sucrose, glucose, or lactate)-amended growth medium, which we previously showed maintained the highest taxa diversity representative of an environment in the anterior regions of the maxilla, where food particles may be stuck for longer periods of time and where saliva velocity is low (26, 27). This is a highly understudied environment that is of significant interest due to its association with caries disease. Bacteria were incubated in 1-ml cultures in a 24-well plate setup, and growth could be observed at the bottom of each growth well after an initial incubation period in a blood-based SHI medium (Fig. S1). (For more details on growth conditions, see Materials and Methods.) Spent growth media were collected from each growth well at different time points for LC-MS/MS analysis (Fig. S1). The obtained MS/MS spectra were analyzed by using the GNPS network (22) and DEREPLICATOR (23) infrastructures, which were previously developed to analyze large MS/MS data sets where one sample may contain several thousand MS/MS spectra (Fig. 1). Currently, GNPS (http://gnps.ucsd.edu/) (22) has approximately 100 million MS/MS spectra available for analysis, of which 7.7 million have matches (dereplicated) to 15,477 known compounds (28). Additional features in GNPS include the annotations of theoretical masses for the most common adducts M+H, M+H2, M+K, and M+Na (i.e., variants of the same compound but with direct additions of new molecules). DEREPLICATOR enables high-throughput PSM identification that is compatible with large-scale mass spectrometry-based screening platforms. The tool constructs theoretical spectra for all peptides in chemical structure databases (e.g., PubChem and AntiMarin), which enables PSMs to be dereplicated without reference spectra. To demonstrate the power of DEREPLICATOR, Mohimani and colleagues (23) analyzed the approximately 100 million spectra in GNPS and identified twice as many PSMs. Based on these findings, we employed both infrastructures to improve putative annotation of mass spectra in this study. Despite the eventual influence of adducts, the high number of PSMs (400 to 900 parent masses per bacterial isolate and time point of growth) that we observed here is noteworthy (see discussion below). Discussions of annotations of ion fragmentation spectra (MS/MS) from mass spectrometry experiments in the following sections follow the “level 2” annotation standard described by the metabolomics standard initiative (29). This standard was based upon significant literature, which decidedly supports putative identification of SMs based on their ion fragment spectra (30–36). Also, most of the annotations that were identified here matched benchmarked compounds that were obtained from isolated or commercial standards in house: therefore, we feel more confident assigning their putative annotations. In addition, to minimize effects of false positives, such as adducts in our comparative data analysis, we only included SMs that could be identified in replicate samples.

10.1128/mSystems.00058-17.1 FIG S1  Experimental setup for growth of bacterial isolates and the *in vitro* biofilm in a 24-well format. Red wells (16 SHI) correspond to the first period of incubation for 16 h in SHI medium and sucrose (2.7 mM). Some isolates did not show any visible growth until later and were therefore incubated for longer periods of time (40 and 68 h). Blue wells correspond to the second part of the incubation, where SHI medium had been removed and biofilms were washed and incubated in a minimal cdm and glucose (2.7 mM [lactate for *Veillonella* species]). The time point shown above each column (from 5 h to 96 h) represents when samples were collected. Transparent wells indicate that no sample was collected. For the time series study of the *in vitro* biofilm, samples were grown in SHI medium only and collected at other time intervals than those shown here (see Materials and Methods). All samples in this study were grown as replicates (not shown in the figure). Download FIG S1, PDF file, 1.1 MB. Copyright © 2017 Edlund et al. 2017 Edlund et al. This content is distributed under the terms of the Creative Commons Attribution 4.0 International license.

### PSM production over time during sugar fermentation.

**FIG 2:**
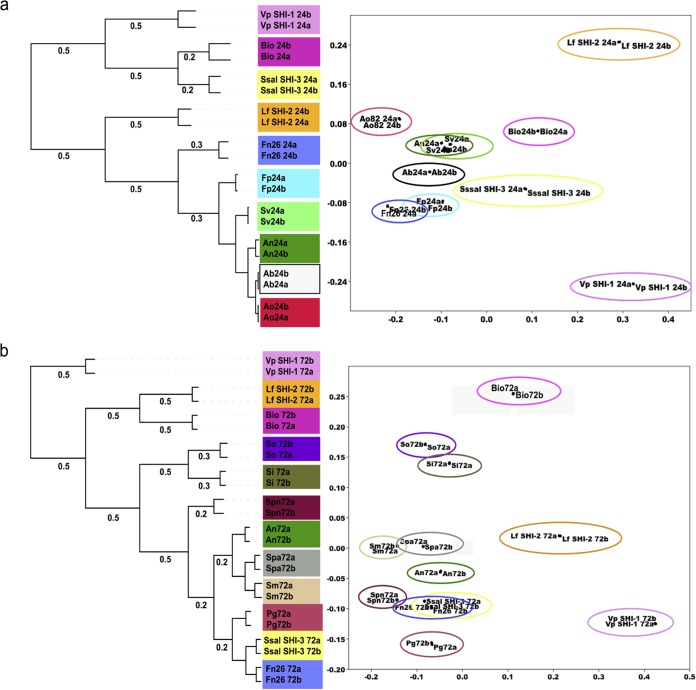
Hierarchical cluster analysis and multidimensional scaling (MDS) analysis of replicate parent mass profiles of growth extracts from oral isolates and the *in vitro* biofilm community from liquid chromatography mass spectrometry (LC-MS). The tree topography shows Pearson correlation distance measures at tree branches (left panel). MDS ordinations for each time point are presented in the right panel. (a) Clustering of replicate growth extracts (a and b) from bacterial isolates and the *in vitro* biofilm community (Bio24a and -b) at 24 h of growth. (b) Clustering of replicate growth extracts (a and b) from bacterial isolates and the *in vitro* biofilm community (Bio72a and -b) at 72 h of growth. Abbreviations: Ab, *Actinomyces bovis*; An, *Actinomyces naeslundii*; Ao, *Actinomyces odontolyticus*; Bio, *in vitro* biofilm community; Fn, *Fusobacterium nucleatum*; Fp, *Fusobacterium periodonticum*; Lf, *Lactobacillus fermentum* SHI-2; Pg, *Porphyromonas gingivalis*; Si, *S. infantis*; Spn, *S. pneumoniae*; Sv, *S. vestibularis*; Sm, *S. mitis*; So, *S. oralis*; Ss, *S. salivarius* SHI-3; Spa, *S. parasanguinis*; Vp, *Veillonella parvula* SHI-1.

In a previous global genome mining study of thousands of genomes from bacterial isolates of the human microbiota and 752 metagenomes from five human body sites, 3,118 SM biosynthetic gene clusters (BGCs) were identified (37). The oral cavity was by far the richest environment (together with the gut) and was found to harbor 1,061 BGCs (37). Products of these BGCs are for the most part unknown, and the elucidation of these remains a daunting challenge for the understanding of key ecological functions of the human microbiome. Larger BGCs, such as those encoding polyketides (PKs) and nonribosomal peptides (NRPs), are often horizontally transferred between closely related strains and species, and therefore, bacterial phylogenetic signatures have been associated with these in previous studies (38, 39). In this study, we applied hierarchical cluster analyses (using Pearson correlation) and multidimensional scaling (MDS) ordinations to capture relationships between MS/MS spectra at two growth stages (at 24 and 72 h of growth, respectively). We chose to incubate cultures for longer periods of time since secondary metabolites are known to be produced during the stationary phase (later stages of growth). Our previous findings from the *in vitro* biofilm community also showed that no significant cell division activity (i.e., growth) occurred during incubation under similar growth conditions (20). However, overall metabolic activity (gene transcription and metabolic output [i.e., primary and secondary metabolites]) remained high, indicating that most bacteria in the biofilms had entered the stationary phase (20). The cluster analyses revealed a significant difference (two-way analysis of variance [ANOVA] and Tukey’s multiple comparisons test, with *P* values ranging between ≤0.001 and ≤0.05) between bacterial species and time points (Fig. 2; see Table S2 and Table S3 posted at ftp://massive.ucsd.edu/MSV000079151/updates/2017-06-30_aedlund_64677506/other/). Notably, the reproducibility was high between replicate MS/MS profiles obtained from separate growth cultures representative of the same species (Fig. 2; see Fig. S2 in the supplemental material). A few examples of species-specific MS/MS signatures were observed for *S. parasangunis* and *L. fermentum* SHI-2, as their PSM profiles from two different time points clustered together for respective species. At 24 h of growth, *Streptococcus salivarius* SHI-3 and *Veillonella parvula* SHI-1 clustered most closely to the species complex biofilm community from which they were originally isolated (Fig. 2) (30). These two species also showed concurrently high gene transcription activity at the genome level in low pH in a previous study (20). To address to what extent *V. parvula* SHI-1, *S. salivarius* SHI-3, and the *in vitro* biofilm community produced similar PSMs, we conducted GNPS MS/MS network analysis of ion fragment spectra from monocultures, including the two species and the *in vitro* biofilm. This revealed a total of 2,151 MS/MS features, of which the biofilms shared 418 MS/MS features (~20%) with *S. salivarius* SHI-3, 250 with *V. parvula* SHI-1 (~12%), and 233 (~11%), with both *S. salivarius* SHI-3 and *V. parvula* SHI-1, while 362 MS/MS (~17%) features were unique (see Table S4 posted at ftp://massive.ucsd.edu/MSV000079151/updates/2017-06-30_aedlund_64677506/other/). These results demonstrate that it is potentially feasible to use MS/MS spectra derived from monocultures of bacterial species to putatively annotate species-unique PSMs in highly complex and understudied biofilm communities. However, a limitation to this approach is that bacteria can have different physiologies when grown as planktonic versus biofilm or monospecies versus multispecies, and therefore they may produce conditionally specific PSMs leading to low overlap. Regardless of this discrepancy, our results suggest that cultivated lab strains of bacteria can serve as a guide to putatively annotate PSMs taxonomically in complex microbial communities, such as oral biofilms. Such annotation approach can have important applications in future disease diagnostics to verify presence of specific pathogens. A cometabolic relationship between oral *Veillonella* and *Streptococcus* was previously identified in dual-species interaction studies where *Veillonella* uses *Streptococcus*-produced lactate as the sole carbon source (40). However, recent studies suggest that we are only just beginning to understand this relationship as dual-species biofilms of the two are less susceptible to antimicrobial treatments than individual monoculture biofilms, suggesting a more complex metabolic interaction (41). Based on these previous studies, our findings here, and our previous metatranscriptomics study of the same *in vitro* biofilm model system (20), we suggest that a specific interaction between *S. salivarius* SHI-3 and *V. parvula* SHI-1 is represented by cometabolic interactions at the level of PSMs. In conclusion, by using the applied clustering approaches, no clear phylogenetic congruency could be revealed from MS/MS profile comparisons (e.g., *Streptococcus* species did not form a separate cluster). Therefore, we suggest that the overall production of PSMs in this study may reflect PSM biosynthesis via minor enzymatic modifications (e.g., methylation, acetylation, etc.) of peptides and amino acids and not biosynthesis via complex PK and NRP gene clusters with phylogenetic signatures. The overall presence of PSMs with no phylogenetic signature is also in line with our previous metatranscriptome study of the complex oral *in vitro* biofilm model system (20), where activity changes of the genes and pathways (KEGG orthology level) were not in total agreement with phylogeny in response to carbohydrate amendment and pH stress, e.g., *S. agalactiae*’s differentially expressed pathways clustered closely with *L. fermentum* SHI-2 while *S. parasangunis* activities clustered with *Klebsiella* sp. and not with other *Streptococcus* species. To better understand the phylogenetic relatedness of PSMs in future studies, a larger number of bacterial isolates from major taxonomic groups, grown in different growth media under different growth conditions, should be considered.

10.1128/mSystems.00058-17.2 FIG S2  Hierarchical cluster analysis of replicate ion fragment (MS/MS) profiles of growth extracts from oral isolates and the *in vitro* biofilm community from liquid chromatography-mass spectrometry (LCMS). The tree topography shows Pearson correlation distance measures at tree branches. Replicate growth extracts (a and b) from bacterial isolates and the *in vitro* biofilm community were obtained from 24 and 72 h of growth, respectively. Download FIG S2, PDF file, 0.2 MB. Copyright © 2017 Edlund et al. 2017 Edlund et al. This content is distributed under the terms of the Creative Commons Attribution 4.0 International license.

### A broad diversity of secreted PSMs is revealed across time.

**FIG 3:**
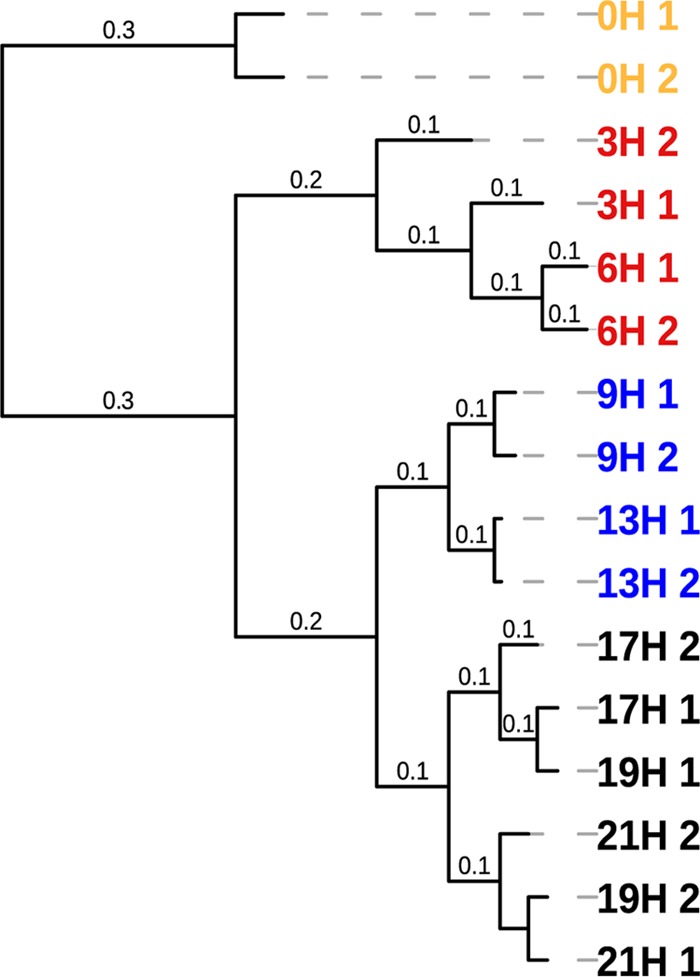
Hierarchical cluster analysis of reproducible parent masses (MS) representative of produced SMs from oral *in vitro*-grown biofilm representing >100 bacterial species. MS profiles from replicate growth medium extracts were obtained from several growth stages: from 0 h (the time point when saliva was inoculated into SHI medium) to 21 h of incubation in SHI medium and sucrose. Pearson correlation distances are shown at tree branches. Distinct clusters of MS profiles are shown as colored leaves on the tree. Yellow leaves, 0 h of growth (0H) with saliva inoculated in SHI medium and sucrose; red leaves, 3 and 6 h of growth (3H and 6H, respectively); blue leaves, 9 and 13 h of growth (9H and 13H, respectively); black leaves, 17, 19, and 21 h of growth (17H, 19H, and 21H, respectively).

**FIG 4:**
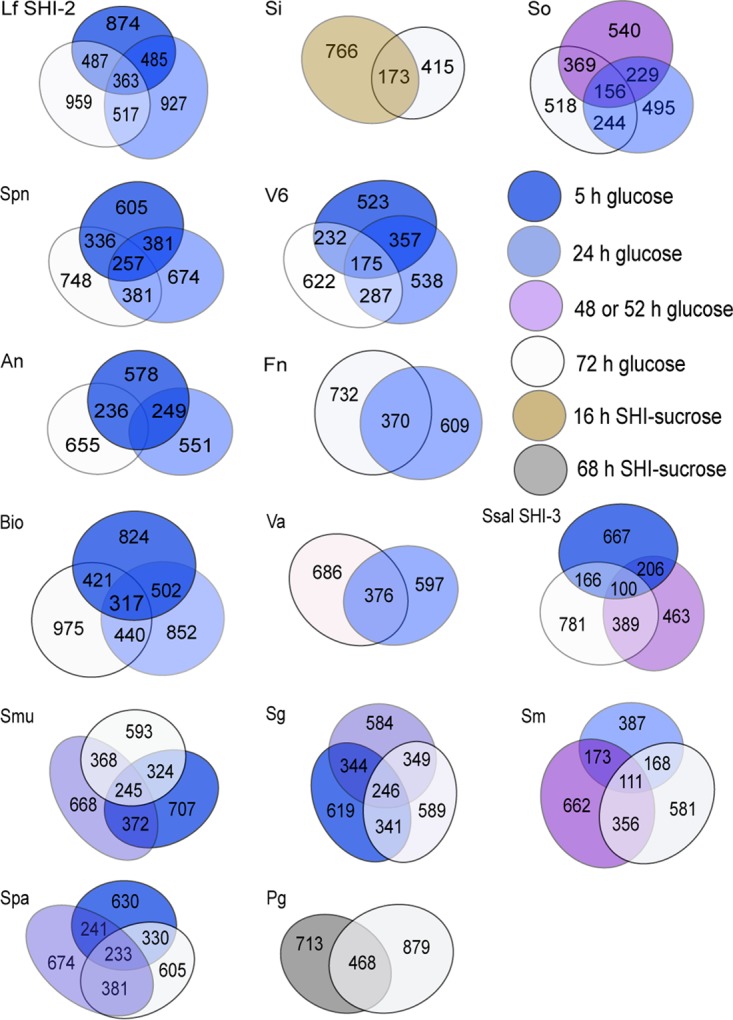
Venn diagrams showing unique and shared molecular masses produced at different growth stages of *in vitro* biofilms (Bio) and 14 of the total 27 studied bacterial isolates. Abbreviations: An, *Actinomyces naeslundii*; Fn, *Fusobacterium nucleatum*; Lf, *Lactobacillus fermentum*; Pg, *Porphyromonas gingivalis*; Sg, *S. gordonii*; Si, *S. infantis*; Sm, *S. mitis*; Smu, *S. mutans*; So, *S. oralis*; Spa, *S. parasanguinis*; Spn, *S. pneumoniae*; Ssal, *S. salivarius* SHI-3; Va6, *Veillonella* sp. strain 6127; and Vp, *Veillonella parvula*. See Fig. S1 for detailed strain abbreviations. Number of parent masses are reported for time points 5, 24, 48 or 52, and 72 h.

By applying a comparative network approach of ionized parent mass (MS/MS) profiles from different time points of growth, we could identify that bacteria secrete a wider variety of PSMs than when analyzing a single time point (Fig. 3 and 4), which suggests that natural product discovery endeavors may benefit from screening multiple growth stages of bacterial isolates. Approximately 400 to 900 PSMs with MS/MS profiles were produced by each isolate per time point (Fig. 4). Comparative MS/MS analyses of samples that contain a high diversity of metabolites is not an exact quantitative measure due to the fact that some parent masses may not be selected for collision-induced dissociation (CID). In addition, the method we applied here specifically targets peptidic SMs within the size range of approximately 100 to 2,000 Da, which excludes many other SMs. In this study, to circumvent loss of parent masses that do not disassociate, we applied a stepping approach that allowed us to apply collision energy stepping coupled with TOF transfer stepping, which is known to provide thorough fragmentation of a diversity of molecules in a single LC-MS run (42).

When analyzing all MS/MS profiles together, using the GNPS network tool, only 153 profiles had matching annotations (more details of these annotations are presented in the discussion below) (see Table S1 posted at ftp://massive.ucsd.edu/MSV000079151/updates/2017-06-30_aedlund_64677506/other/), which suggest that most PSMs produced by oral bacteria are unknown. Parent mass distributions of the identified masses varied from *m*/*z* 110 to 1,865 (see Fig. S3 in the supplemental material). Approximately one-third of parent masses ranged between *m*/*z* 110 and 299, while the remaining masses ranged between *m*/*z* 300 and 899. Only a smaller fraction was of larger sizes: *m*/*z* 900 to 1,865 (Fig. S3). An hourly comparison of secreted SMs from the *in vitro* biofilm community revealed a shift in SM profiles starting as early as after 3 h of inoculation (Fig. 3). Relatively similar molecular masses were detected after 6 h; however, clear shifts occurred at 9 and 17 h of biofilm growth (Fig. 3). The implications of this dynamic behavior of PSMs for human health are unknown, which suggests that more emphasis should be put on understanding the role of PSM changes over time. Such information would provide a deeper knowledge of detailed short-term mechanisms that foster overall key functions of microbial communities.

10.1128/mSystems.00058-17.3 FIG S3  Molecular network of MS/MS data collected from 27 oral bacterial isolates and an *in vitro* oral biofilm model. Colors of nodes (circles) represent arbitrary size groups of molecular features (*m*/*z*) as follows: red, *m*/*z* 110 to 299; white, *m*/*z* 300 to 499; turquoise, *m*/*z* 500 to 699; blue, *m*/*z* 700 to 899; yellow, *m*/*z* 900 to 1,865. Download FIG S3, PDF file, 0.2 MB. Copyright © 2017 Edlund et al. 2017 Edlund et al. This content is distributed under the terms of the Creative Commons Attribution 4.0 International license.

### GNPS and DEREPLICATOR annotation pipelines reveal PSM candidates with structural similarities to compounds with known bioactivity.

By analyzing all obtained MS/MS spectra representative of the 27 bacterial species using GNPS network (22) and DEREPLICATOR approaches (23), we were able to identify putative analogs of PSMs belonging to known classes of compounds, which will be discussed here. It is important to highlight that even small changes in chemical structure (here viewed as differences in ion fragments between query MS/MS spectra and matching benchmark MS/MS spectra) can greatly impact biological function. Since we rely on automated annotation and a level 2 classification standard to identify PSMs, the following discussion regarding their biological role is not absolute.

Our previous study of the oral *in vitro* biofilm model system indicated the presence of PSMs such as single amino acid derivatives, dipeptides, and lactone-like compounds during growth at low pH (20), and therefore we choose to explore their production in more detail here. Results from GNPS networks revealed a total of 32 putative dipeptide annotations (see Table S1 posted at ftp://massive.ucsd.edu/MSV000079151/updates/2017-06-30_aedlund_64677506/other/). These were structurally similar to 13 known peptides with defined MS/MS profiles in the GNPS database (cyclo-Gly-Leu, cyclo-Leu-Pro, cyclo-Pro-Val, cyclo-Leu-Phe, cyclo-Pro-Gly, cyclo-Thr-Pro, cyclo-Val-Phe, cyclo-Ala-Leu, cyclo-Phe-4-Hyp, cyclo-Phe-Pro, cyclo-Trp-Pro, cyclo-Tyr-Pro, cyclo-Val-Pro) (see Table S1 posted at the above URL). The analysis showed a mixed taxonomic origin of these peptides (i.e., several phylogenetically distinct bacterial isolates were able to produce them). Although the functional role of most of these dipeptides is unknown, a few earlier studies showed they have critical roles in regulating the production of homoserine lactones (43, 44). Other studies identified that they are capable of interacting with other bacterial community members at the level of gene transcription as well as regulating bacterial population sizes and survival (45, 46).

**FIG 5:**
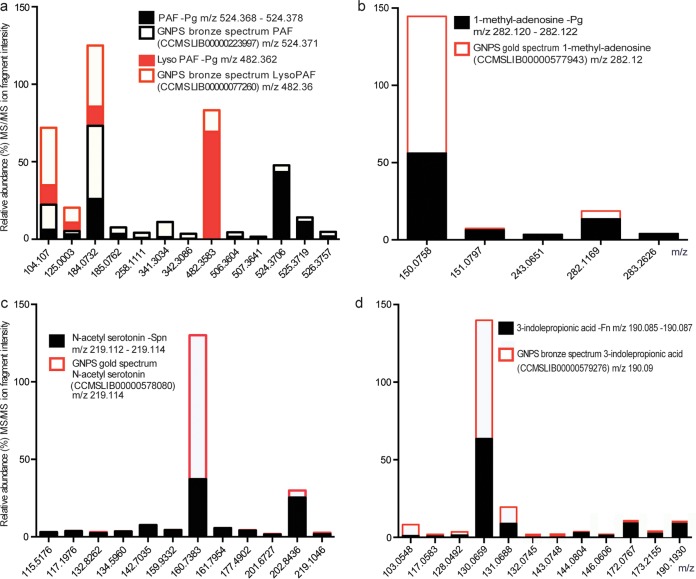
Comparisons of MS/MS ion fragmentation spectra derived from GNPS network annotations (see Table S4 posted at ftp://massive.ucsd.edu/MSV000079151/updates/2017-06-30_aedlund_64677506/other/) and from the GNPS library of benchmarked compounds. The *x* axis shows fragment ion peaks (*m*/*z*), and the *y* axis shows the percentage of relative abundance MS/MS ion fragment intensity. (a) MS/MS ion spectra showing major matching ion peaks for platelet activating factor (PAF) (parent mass for growth extract spectrum, *m*/*z* 524.368 to 534.378; parent mass for GNPS bronze spectrum, PAF, *m*/*z* 524.371) and the PAF precursor lyso-PAF (parent mass for growth extract spectrum, *m*/*z* 482.362; parent mass for GNPS bronze spectrum lyso-PAF, *m*/*z* 482.36). (b) MS/MS ion spectra showing major matching ion peaks for 1-methyladenosine (parent mass for growth extract spectrum, *m/z* 282.120 to 282.122; parent mass for GNPS bronze spectrum 1-methyladenosine, *m*/*z* 282.12). (c) MS/MS ion spectra showing major matching ion peaks for *N*-acetylserotonin (parent mass for growth extract spectrum, *m/z* 219.112 to 219.114; parent mass for GNPS gold spectrum *N*-acetylserotonin, *m*/*z* 282.12). (D) MS/MS ion spectra showing major matching ion peaks for 3-indolepropionic acid (parent mass for growth extract spectrum, *m/z* 190.085 to 190.087; parent mass for GNPS bronze spectrum 3-indolepropionic, *m/z* 219.085).

The previously identified PKS-NRPS metabolite mutanobactin A (*m/z* 721.4350), which was isolated from *S. mutans* UA159 (14), was also identified at all growth stages in *S. mutans* UA159 biofilms here (see Tables S1 and S5 at the above URL). Ion fragments from the growth extracts were compared to the pure compound (*m*/*z* 721.4380), which was available in our lab as a gift from the Qi lab (14) (see Table S5 at ftp://massive.ucsd.edu/MSV000079151/updates/2017-06-30_aedlund_64677506/other/). Both GNPS and DEREPLICATOR were able to identify this PSM, showing that our experimental protocols were optimized for extraction and identification of such PKS-NRPS metabolites (see Tables S5 and S6 at the above URL). Other interesting putative PSMs, produced both by bacterial isolates and the *in vitro* biofilm community, were platelet activating factor (PAF) C-16 (*m*/*z* 524.368 to 524.378) and lyso-PAF (*m*/*z* 482.362 to 482.362)-like compounds (Fig. 5a; see Table S1 posted at the above URL). PAF-like PSMs could be identified in growth extracts from multiple bacterial species belonging to the genera *Fusobacterium*, *Streptococcus*, and *Actinomyces* and the periodontal pathogen *Porphyromonas gingivalis*. A lyso-PAF-like PSM was only identified in *P. gingivalis* growth extracts (Fig. 5a; see Table S1 posted at the above URL). PAF is a potent lipid mediator with various biological activities, including platelet and leukocyte activation. It is produced by eukaryotes; however, the final step in its biosynthesis (i.e., the conversion of lyso-PAF to PAF) has also been observed to be carried out by various bacterial strains, e.g., *Escherichia coli* (*E. coli*) K-12 (47, 48), as well as *Salmonella enterica* serovar Typhimurium and *Helicobacter pylori* (49). Since a blood-based medium was used to seed each bacterial culture in this study, it is possible that PAF was synthesized from medium-derived lyso-PAF. PAF was not identified in the growth medium controls, and therefore our results support earlier findings that oral bacteria can convert eukaryotic lyso-PAF to PAF, which could play additional roles in platelet aggregation and inflammation. To verify that bacteria can conduct the last step of PAF synthesis in future studies, isotope-labeled lyso-PAF could be used as a substrate and traced through metabolic pathways in monocultures of bacterial members from the human microbiome.

Another putative PSM that was identified by the GNPS network analysis, from growth extracts of the periodontal pathogen *P. gingivalis*, was a 1-methyladenosine (m1a)-like molecule (*m*/*z* 282.120 to 282.122) (Fig. 5b; see Table S1 at the above URL). This modified nucleoside is known as major mRNA and DNA modifier in both eukaryotic and prokaryotic cells (50). It occurs on thousands of different transcripts in eukaryotic cells at an estimated average transcript stoichiometry of 20% in humans (51). It responds to physiological conditions and correlates positively with protein production, which indicates a strong functional role of m1a in promoting translation of methylated mRNA (52). Compared to regular adenosine, m1a has an additional methyl group at the Watson-Crick interface, and due to this structural change, m1a cannot only lead to truncated cDNA but can also cause misincorporation at the site of read-through of cDNA (50). Based on these findings and the fact that *P. gingivalis* has the potential to invade human cells (e.g., oral epithelial cells), it is possible that *P. gingivalis*-secreted nucleoside can interfere with the human DNA and RNA machinery. The role of bacterially produced nucleosides in eukaryotic DNA and RNA synthesis is completely unexplored and could be further investigated by studying human intracellular pathogens (e.g., species belonging to *Fusobacterium*, *Porphyromonas*, and the *Chlamydia*/*Chlamydophila* group).

An *N*-acetylserotonin-like molecule (*m*/*z* 219.112 to 219.115) was identified in multiple oral isolates belonging to the *Streptococcus* genus (i.e., *S. sanguinis*, *S. mutans*, *S. salivarius* SHI-3, and *S. pneumoniae*) (Fig. 5c; see Table S1 posted at ftp://massive.ucsd.edu/MSV000079151/updates/2017-06-30_aedlund_64677506/other/). *N*-Acetylserotonin is an intermediate in melatonin production and is now recognized as ubiquitous among living organisms, including humans, animals, plants, bacteria, fungi, and macroalgae (53–56). *N*-Acetylserotonin was recently shown to be produced by both photosynthetic bacteria and endophytic bacteria from grapevine roots (57, 58). In the latter study, the bacterium *Bacillus amyloliquefaciens* SB-9 exhibited the highest level of *in vitro* melatonin secretion and also produced three intermediates of the melatonin biosynthesis pathway: 5-hydroxytryptophan, serotonin, and *N*-acetylserotonin (59). Our study is the first to observe an *N*-acetylserotonin-like molecule produced from oral bacterial community members. This is a particular interesting finding as it indicates that the human oral microbiome could possibly impact hormonal levels related to human mood and sleeping patterns. Interactions between bacterial *N*-acetylserotonin-like production and human cells could be tested in future experiments, and *in vivo* production can be monitored in saliva and plaque samples.

GNPS network analysis also identified a putative 3-indolepropionic acid (IPA)-like molecule (*m*/*z* 190.085 to 190.087) in growth extracts from various isolates belonging to the *Streptococcus*, *Lactobacillus*, *Actinomyces*, and *Fusobacterium* genera (Fig. 5c; see Table S1 posted at the above URL). IPA is already known to be produced by the human microbiome, which is interesting since it has shown neuroprotective abilities (60, 61) and is an even more potent scavenger of hydroxyl radicals than melatonin, the most potent scavenger of hydroxyl radicals synthesized by human enzymes (47). Similar to melatonin but unlike other antioxidants, IPA scavenges radicals without subsequently generating reactive and prooxidant intermediate compounds (48, 49). To further elucidate PSM annotations of the obtained MS/MS spectra, we also analyzed all MS/MS spectra with the DEREPLICATOR infrastructure (23). DEREPLICATOR compares mass spectra to predicted spectra of peptide natural products obtained from available structure-based databases (e.g., PubChem and AntiMarin) and is therefore also based on putative annotations that need experimental validation for exact identification. In this study, DEREPLICATOR provided a list of annotated molecules (see Table S6 at the above URL), which was sorted based on matching score and false discovery rate (FDR). Six parent masses showed annotations that were significant (*P* < 0.001) with FDR values of 0% (see Table S6). As previously mentioned, mutanobactin A (*m*/*z* 720.424) could be identified as being secreted from *S. mutans* UA159. A putative BZR-cotoxin II (*m*/*z* 964.573), earlier identified from endophytic fungi (62), was also identified in growth extracts from *Streptococcus gordonii* ATCC 10558 (FDR, 0%). Moreover, two cyclic peptides (i.e., putative annotations eptidemanamide [*m/z* 853.383] and anacyclamide A10 [*m/z* 1,052.53]), were also identified from growth extracts of *Streptococcus pneumoniae* TCH8431 and *S. sangunis* VMS66, respectively. Also, a putative l-valyl-l-leucyl-l-prolyl-l-valyl-l-prol peptide (*m*/*z* 652.408; rt, 215.65) was identified in 52 different growth extracts representative of bacterial isolates of various taxonomic origins (i.e., *Veillonella*, *Streptococcus*, and *Actinomyces*) (see Table S6 at the above URL). Similar results were obtained in GNPS, where this PSM was identified in 41 different spectra (*m*/*z* 652.407; rt, 215.650). Future research to identify and annotate this “unknown” PSM needs to be conducted to gain a deeper understanding of its role in oral microbial ecology. We also employed the VarQuest algorithm in DEREPLICATOR to search for analogues of known natural products in the obtained MS/MS data. This resulted in identification of additional putative PSMs from growth extracts of *S. pneumonia*, *S. gordonii*, *Streptococcus vestibularis*, and *Streptococcus sobrinus*, which showed high homology to hymenamide H (*m*/*z* 903.52), callyaerin H (*m*/*z* 1,043.62), axinastatin 4 (*m*/*z* 806.47), gramicidin S2 (*m*/*z* 1,126.69), phakellistatin 11 (*m*/*z* 973.51), janthinocin A (*m*/*z* 1,192.61), micropeptin C (*m*/*z* 1,027.49), and Sch 378167 5′,5″-diamide (*m*/*z* 1,135.66) (as shown on the GNPS site at http://gnps.ucsd.edu/ProteoSAFe/result.jsp?task=244733240c5649e4a46216fda4e064ec&view=view_significant_unique#%7B%22main.FDR_upperinput%22%3A%221%22%2C%22table_sort_history%22%3A%22main.P-Value_asc%22%7D).

In conclusion, by using a comparative analysis approach based on the annotation of parent masses in growth cultures of individual oral bacterial isolates in parallel with the highly complex *in vitro* biofilm community, we identified unique signatures of PSMs over time, which suggests that the oral microbiome has a highly dynamic chemotype that is potentially involved in the regulation of community succession and bacterium-to-bacterium interactions as well as cell-to-host signaling. As of today, little information exists on the role and the underlying drivers of the differential production of PSMs, and the fact that only a few of the produced PSMs (2.2%) (see Table S1 posted at ftp://massive.ucsd.edu/MSV000079151/updates/2017-06-30_aedlund_64677506/other/) could be putatively annotated highlights that this area of microbiome research remains a black box and needs significant attention to provide a deeper understanding of key interactions between the human host and its microbiome. The GNPS and DEREPLICATOR annotation pipelines allowed us to identify patterns in metabolite production that can be linked to specific taxonomic units and culture conditions. Furthermore, these annotation approaches also provided a survey of the biosynthetic capacity of common oral bacterial community members and a method to compare isolates based on the variety of their SMs.

## MATERIALS AND METHODS

### Description of growth media, bacterial strains, and saliva inoculum.

Chemically defined medium (cdm) was modified after previous protocols (51, 53). SHI medium was prepared after the protocol of Tian et al. (54). Detailed cdm and SHI medium preparation protocols are available at http://depts.washington.edu/jsmlab/downloads/protocols/. The following bacterial strains were obtained from the American Type Culture Collection (ATCC) and included in this study: *Actinomyces bovis* ATCC 1368, *Actinomyces meyeri* ATCC 35568, *Actinomyces naeslundii* OMZ 724, *Actinomyces odontolyticus* ATCC 17929, *A. odontolyticus* ATCC 17982, *Actinomyces viscosus* ATCC 15987, *Fusobacterium nucleatum* 23726, *F. nucleatum* oral taxon 420, *Fusobacterium periodonticum* 1A 54-D1, *Streptococcus gordonii* ATCC 10558, *Streptococcus infantis* HOT-638, *Streptococcus mitis* bv2 strain ATCC F0392, *Streptococcus mutans* UA159, *Streptococcus oralis* HOT-707, *Streptococcus parasanguinis* ATCC 15911, *Streptococcus pneumoniae* TCH8431, *Streptococcus sanguinis* VMC66, *Streptococcus sobrinus* OMZ177, *Streptococcus* sp. strain C150, *Streptococcus vestibularis* F0396, *Porphyromonas gingivalis* F0568, *Veillonella* sp. strain 6127, and *Veillonella* oral taxon 158 strain F0412. The following oral bacterial isolates were obtained from our own research laboratory: *Actinomyces* sp. strain XH001 (8), *Lactobacillus fermentum* SHI-2, *Veillonella parvula* SHI-1, and *Streptococcus salivarius* SHI-3. Species identity was verified for each isolate by sequencing of the 16S rRNA-encoding gene at the Genewiz, Inc. (La Jolla, CA), sequence facility using the fD1-16S rRNA primer (55) encompassing approximately 600 bp of the gene. A saliva inoculum for *in vitro* biofilm growth was collected and pooled from six healthy subjects (ages 25 to 35 years) as described by Edlund and colleagues (19).

### Incubation conditions for oral bacterial isolates and saliva-derived *in vitro* biofilms.

Individual isolates (except those belonging to the *Veillonella* genus) and pooled saliva inoculum (19) were seeded into separate replicate growth wells (2 replicates per isolate or inoculum) in SHI medium and sucrose (0.5%) within sterile 24-well plates (20). Members belonging to the *Veillonella* genus that cannot metabolize sucrose were seeded in lactate as a carbon source. Cell-free saliva was used for coating wells prior to growing biofilms (20). After 16 h of growth in 37°C in anaerobic conditions, SHI medium was carefully removed from each growth well and the bottom of each well was screened with the naked eye for biofilm formation. For cultures in which no visible biofilms had formed at this time point, incubation continued for another 24 or 52 h (see SHI 40 and 68 h in Fig. S1 at ftp://massive.ucsd.edu/MSV000079151/updates/2017-06-30_aedlund_64677506/other/) prior to continuing with the remaining biofilm wash and carbohydrate amendment steps. When biofilm establishment was confirmed and SHI medium had been removed, extra careful washing of the biofilms with buffered chemically defined medium (cdm) was performed. After the washing step, biofilms were starved in fresh cdm (pH 7) for 2 h in 37°C under anaerobic conditions. After 2 h of starvation, the spent cdm was carefully removed, and 1 ml of fresh cdm (pH 7) supplemented with either glucose (0.5%) or lactate (27.8 mM) was added to each growth well.

### Sample collection and organic solvent compound extraction for LC-MS/MS analyses.

For each sample presented in Fig. S1, replicate biofilm samples were collected from two growth wells for organic solvent extraction in separate sterile 2.0-ml Eppendorf tubes. Samples were immediately frozen on dry ice inside in the anaerobic chamber and then transferred to −80°C storage until organic solvent extraction. PSMs were extracted by using the following protocol: ethyl acetate (3:1 ratio) was added to each frozen sample (final volume, 1,200 µl). Directly after thawing, samples were resuspended by pipetting and sonication at maximum frequency for 10 min in a water bath followed by incubation in room temperature for 15 min. Samples were then centrifuged at 14,000 rpm for 5 min. Supernatants were transferred to clean tubes and dried under vacuum in a lyophilizer. When completely dry, 1 volume of acetonitrile and methanol (1:1 ratio) was added to each of the dried extracts, which were resuspended by vortexing, and a second round of sonication, incubation at room temperature, and centrifugation was performed, as described above. After the last centrifugation step, sample extracts were concentrated and dried under vacuum in a lyophilizer. Concentrated samples were stored at −80°C until they were ready to be analyzed by LC-MS/MS.

### LC-MS/MS analysis.

For LC-MS/MS analysis, the dried samples were dissolved in 150 µl 80% methanol and diluted 100-fold. The resuspended extracts were analyzed with an UltiMate 3000 ultrahigh-performance liquid chromatography (UHPLC) system (Thermo Fisher Scientific, Carlsbad, CA) using a Kinetex 1.7-µm C_18_ reversed-phase UHPLC column (50 by 2.1 mm) and a Maxis qTOF mass spectrometer (Bruker Daltonics, Billerica, MA) equipped with an electrospray ionization (ESI) source. The chromatography was performed at a flow rate of 0.5 ml/min throughout the run. MS spectra were acquired in positive-ion mode in the mass range of *m*/*z* 100 to 2,000. An external calibration with ESI-L low-concentration tuning mix (Agilent Technologies, La Jolla, CA) was performed prior to data collection, and internal calibrant hexakis(1*H*,1*H*,3*H*-tetrafluoropropoxy)phosphazene was used throughout the runs. The capillary voltage of 4,500 V, nebulizer gas pressure (nitrogen) of 160 kPa, ion source temperature of 200°C, dry gas flow of 7 liters/min at source temperature, and spectral rate of 3 Hz for MS^1^ and 10 Hz for MS^2^ were used. To acquire MS/MS fragmentation, the 10 most intense ions per MS^1^ were selected. Basic stepping function was used to fragment ions at 50% and 125% of the collision-induced dissociation (CID) calculated for each *m*/*z* (56) with a timing of 50% for each step. Similarly, basic stepping of collision radio frequency (RF) of 550 and 800 V peak to peak (Vpp) with a timing of 50% for each step and transfer time stepping of 57 and 90 µs with a timing of 50% for each step was employed. The MS/MS active exclusion parameter was set to 3 and was released after 30 s. The mass of internal calibrant was excluded from the MS^2^ list.

### Mapping PSMs in GNPS and DEREPLICATOR.

Molecular networking analyses were performed at the UCSD-hosted Global Natural Products Social Molecular Networking web server (http://gnps.ucsd.edu/) (22). This platform provides an overview of the molecular features in mass spectrometry-based metabolomics by comparing ion fragmentation patterns to identify chemical relationships. This comparison is based upon the similarity cosine scoring of MS/MS spectra and the visualization of those relationships in a 2-dimensional network in the Cytoscape software v.3.4.0 (63). A single chemical species is represented as a node, and the relatedness between spectra is represented as an edge. Molecular network analysis was performed separately on raw mxXML files obtained from growth medium extracts of individual bacterial isolates. The *in vitro* biofilm time series (0 to 21 h of growth) was analyzed in network analysis separately. The following network settings were applied: minimum cosine setting, 0.5; Network TopK, 10; minimal matched peaks, 3; minimum cluster size, 2. Run MS cluster was selected. Parent mass tolerance was set to 0.02 Da, and fragment ion mass tolerance was set to 0.02 Da. GNPS analysis parameters, networking statistics, and network summarizing graphs are available on the GNPS site at https://gnps.ucsd.edu/ProteoSAFe/result.jsp?task=48e75e72f8294f25b947a4ff5e828249&view=view_all_clusters_withID_beta.

The DEREPLICATOR program (23), a peptidic natural product workflow, was also employed to compare experimental MS/MS spectra against chemical structure databases: e.g., PubChem and AntiMarin. The following settings were used for DEREPLICATOR analysis: precursor and fragment ion mass tolerance, 0.02 Da; maximum charge, 3; and accurate *P* values, “yes.” All other parameters were set to the default values. We also employed the VarQuest algorithm to search for analogues of known natural products in the obtained MS/MS data. The following running parameters were used: precursor and fragment ion mass tolerance, 0.02 Da; maximum charge, 2; maximum allowed modification mass, 150 Da; minimum matched peaks with known compound, 4; and accurate *P* values, “yes.” All other parameters were set to the default values.

### Cluster analysis.

To identify MS/MS similarities between bacteria and growth stages, hierarchical cluster analysis was applied by using the Multiple Experiment Viewer (MeV) (v.4.8.1) (http://www.tm4.org) with the average linkage setting and Pearson correlation as distance measure. Input matrices sorted on *m/z* values that represented MS profiles from 24 and 72 h of growth were prepared. Only reproducible *m/z* (*m/z* that were identified in replicate samples) were included in the matrix. Another clustering approach, multidimensional scaling from the Past 3.14 software package, was also applied using the same matrices.

### Accession number(s).

All data sets are accessible from the MassIVE repository (https://massive.ucsd.edu/), and the associated accession numbers are MSV000079158 and MSV000079151.

## References

[1] Cho I, Blaser MJ 2012 The human microbiome: at the interface of health and disease. Nat Rev Genet 13:260–270. doi:10.1038/nrg3182. 22411464 PMC3418802

[2] Round JL, Mazmanian SK 2009 The gut microbiota shapes intestinal immune responses during health and disease. Nat Rev Immunol 9:313–323. doi:10.1038/nri2515. 19343057 PMC4095778

[3] Aas JA, Paster BJ, Stokes LN, Olsen I, Dewhirst FE 2005 Defining the normal bacterial flora of the oral cavity. J Clin Microbiol 43:5721–5732. doi:10.1128/JCM.43.11.5721-5732.2005. 16272510 PMC1287824

[4] Mark Welch JL, Rossetti BJ, Rieken CW, Dewhirst FE, Borisy GG 2016 Biogeography of a human oral microbiome at the micron scale. Proc Natl Acad Sci U S A 113:E791–E800. doi:10.1073/pnas.1522149113. 26811460 PMC4760785

[5] Utter DR, Mark Welch JL, Borisy GG 2016 Individuality, stability, and variability of the plaque microbiome. Front Microbiol 7:564. doi:10.3389/fmicb.2016.00564. 27148241 PMC4840391

[6] Yatsunenko T, Rey FE, Manary MJ, Trehan I, Dominguez-Bello MG, Contreras M, Magris M, Hidalgo G, Baldassano RN, Anokhin AP, Heath AC, Warner B, Reeder J, Kuczynski J, Caporaso JG, Lozupone CA, Lauber C, Clemente JC, Knights D, Knight R, Gordon JI 2012 Human gut microbiome viewed across age and geography. Nature 486:222–227. doi:10.1038/nature11053. 22699611 PMC3376388

[7] Kuramitsu HK, He X, Lux R, Anderson MH, Shi W 2007 Interspecies interactions within oral microbial communities. Microbiol Mol Biol Rev 71:653–670. doi:10.1128/MMBR.00024-07. 18063722 PMC2168648

[8] He X, McLean JS, Edlund A, Yooseph S, Hall AP, Liu SY, Dorrestein PC, Esquenazi E, Hunter RC, Cheng G, Nelson KE, Lux R, Shi W 2015 Cultivation of a human-associated TM7 phylotype reveals a reduced genome and epibiotic parasitic lifestyle. Proc Natl Acad Sci U S A 112:244–249. doi:10.1073/pnas.1419038112. 25535390 PMC4291631

[9] Belizário JE, Napolitano M 2015 Human microbiomes and their roles in dysbiosis, common diseases, and novel therapeutic approaches. Front Microbiol 6:1050. doi:10.3389/fmicb.2015.01050. 26500616 PMC4594012

[10] Donia MS, Fischbach MA 2015 Human microbiota. Small molecules from the human microbiota. Science 349:1254766. doi:10.1126/science.1254766. PMC4641445 26206939

[11] Garg N, Luzzatto-Knaan T, Melnik AV, Caraballo-Rodríguez AM, Floros DJ, Petras D, Gregor R, Dorrestein PC, Phelan VV 2017 Natural products as mediators of disease. Nat Prod Rep 34:194–219. doi:10.1039/c6np00063k. 27874907 PMC5299058

[12] McWilliams BD, Torres AG 2014 Enterohemorrhagic Escherichia coli adhesins. Microbiol Spectr doi:10.1128/microbiolspec.EHEC-0003-2013. 26103974

[13] Williams BB, Van Benschoten AH, Cimermancic P, Donia MS, Zimmermann M, Taketani M, Ishihara A, Kashyap PC, Fraser JS, Fischbach MA 2014 Discovery and characterization of gut microbiota decarboxylases that can produce the neurotransmitter tryptamine. Cell Host Microbe 16:495–503. doi:10.1016/j.chom.2014.09.001. 25263219 PMC4260654

[14] Joyner PM, Liu J, Zhang Z, Merritt J, Qi F, Cichewicz RH 2010 Mutanobactin A from the human oral pathogen Streptococcus mutans is a cross-kingdom regulator of the yeast-mycelium transition. Org Biomol Chem 8:5486–5489. doi:10.1039/c0ob00579g. 20852771 PMC2992086

[15] Hyink O, Wescombe PA, Upton M, Ragland N, Burton JP, Tagg JR 2007 Salivaricin A2 and the novel lantibiotic salivaricin B are encoded at adjacent loci on a 190-kilobase transmissible megaplasmid in the oral probiotic strain Streptococcus salivarius K-12. Appl Environ Microbiol 73:1107–1113. doi:10.1128/AEM.02265-06. 17194838 PMC1828679

[16] Reference deleted.

[17] Takahashi N, Schachtele CF 1990 Effect of pH on the growth and proteolytic activity of Porphyromonas gingivalis and Bacteroides intermedius. J Dent Res 69:1266–1269. doi:10.1177/00220345900690060801. 2191980

[18] Bouslimani A, Porto C, Rath CM, Wang M, Guo Y, Gonzalez A, Berg-Lyon D, Ackermann G, Moeller Christensen GJ, Nakatsuji T, Zhang L, Borkowski AW, Meehan MJ, Dorrestein K, Gallo RL, Bandeira N, Knight R, Alexandrov T, Dorrestein PC 2015 Molecular cartography of the human skin surface in 3D. Proc Natl Acad Sci U S A 112:E2120–E2129. doi:10.1073/pnas.1424409112. PMC4418856 25825778

[19] Edlund A, Yang Y, Hall AP, Guo L, Lux R, He X, Nelson KE, Nealson KH, Yooseph S, Shi W, McLean JS 2013 An in vitro biofilm model system maintaining a highly reproducible species and metabolic diversity approaching that of the human oral microbiome. Microbiome 1:25. doi:10.1186/2049-2618-1-25. 24451062 PMC3971625

[20] Edlund A, Yang Y, Yooseph S, Hall AP, Nguyen DD, Dorrestein PC, Nelson KE, He X, Lux R, Shi W, McLean JS 2015 Meta-omics uncover temporal regulation of pathways across oral microbiome genera during in vitro sugar metabolism. ISME J 9:2605–2619. doi:10.1038/ismej.2015.72. 26023872 PMC4817640

[21] Camilli A, Bassler BL 2006 Bacterial small-molecule signaling pathways. Science 311:1113–1116. doi:10.1126/science.1121357. 16497924 PMC2776824

[22] Wang M, Carver JJ, Phelan VV, Sanchez LM, Garg N, Peng Y, Nguyen DD, Watrous J, Kapono CA, Luzzatto-Knaan T, Porto C, Bouslimani A, Melnik AV, Meehan MJ, Liu WT, Crüsemann M, Boudreau PD, Esquenazi E, Sandoval-Calderón M, Kersten RD, Pace LA, Quinn RA, Duncan KR, Hsu CC, Floros DJ, Gavilan RG, Kleigrewe K, Northen T, Dutton RJ, Parrot D, Carlson EE, Aigle B, Michelsen CF, Jelsbak L, Sohlenkamp C, Pevzner P, Edlund A, McLean J, Piel J, Murphy BT, Gerwick L, Liaw CC, Yang YL, Humpf HU, Maansson M, Keyzers RA, Sims AC, Johnson AR, Sidebottom AM, Sedio BE, Klitgaard A, Larson CB, Boya PC, Torres-Mendoza D, Gonzalez DJ, Silva DB, Marques LM, Demarque DP, Pociute E, O’Neill EC, Briand E, Helfrich EJ, Granatosky EA, Glukhov E, Ryffel F, Houson H, Mohimani H, Kharbush JJ, Zeng Y, Vorholt JA, Kurita KL, Charusanti P, McPhail KL, Nielsen KF, Vuong L, Elfeki M, Traxler MF, Engene N, Koyama N, Vining OB, Baric R, Silva RR, Mascuch SJ, Tomasi S, Jenkins S, Macherla V, Hoffman T, Agarwal V, Williams PG, Dai J, Neupane R, Gurr J, Rodriguez AM, Lamsa A, Zhang C, Dorrestein K, Duggan BM, Almaliti J, Allard PM, Phapale P, Nothias LF, Alexandrov T, Litaudon M, Wolfender JL, Kyle JE, Metz TO, Peryea T, Nguyen DT, VanLeer D, Shinn P, Jadhav A, Muller R, Waters KM, Shi W, Liu X, Zhang L, Knight R, Jensen PR, Palsson BO, Pogliano K, Linington RG, Gutierrez M, Lopes NP, Gerwick WH, Moore BS, Dorrestein PC, Bandeira N 2016 Sharing and community curation of mass spectrometry data with Global Natural Products Social Molecular Networking. Nat Biotechnol 34:828–837. doi:10.1038/nbt.3597. 27504778 PMC5321674

[23] Mohimani H, Gurevich A, Mikheenko A, Garg N, Nothias LF, Ninomiya A, Takada K, Dorrestein PC, Pevzner PA 2017 Dereplication of peptidic natural products through database search of mass spectra. Nat Chem Biol 13:30–37. doi:10.1038/nchembio.2219. 27820803 PMC5409158

[24] Kersten RD, Yang YL, Xu Y, Cimermancic P, Nam SJ, Fenical W, Fischbach MA, Moore BS, Dorrestein PC 2011 A mass spectrometry-guided genome mining approach for natural product peptidogenomics. Nat Chem Biol 7:794–802. doi:10.1038/nchembio.684. 21983601 PMC3258187

[25] Grebe SK, Singh RJ 2011 LC-MS/MS in the clinical laboratory—where to from here? Clin Biochem Rev 32:5–31. 21451775 PMC3052391

[26] Van Houte J, Sansone C, Joshipura K, Kent R 1991 Mutans streptococci and non-mutans streptococci acidogenic at low pH, and in vitro acidogenic potential of dental plaque in two different areas of the human dentition. J Dent Res 70:1503–1507. doi:10.1177/00220345910700120601. 1774381

[27] Arneberg P, Giertsen E, Emberland H, Ogaard B 1997 Intra-oral variations in total plaque fluoride related to plaque pH. A study in orthodontic patients. Caries Res 31:451–456. doi:10.1159/000262437. 9353585

[28] Horsman ME, Boddy CN 2016 Natural products: mapping an amazing thicket. Nat Chem Biol 13:6–7. doi:10.1038/nchembio.2265. 27984578

[29] Sumner LW, Amberg A, Barrett D, Beale MH, Beger R, Daykin CA, Fan TW, Fiehn O, Goodacre R, Griffin JL, Hankemeier T, Hardy N, Harnly J, Higashi R, Kopka J, Lane AN, Lindon JC, Marriott P, Nicholls AW, Reily MD, Thaden JJ, Viant MR 2007 Proposed minimum reporting standards for chemical analysis. Chemical Analysis Working Group (CAWG) Metabolomics Standards Initiative (MSI). Metabolomics 3:211–221. doi:10.1007/s11306-007-0082-2. 24039616 PMC3772505

[30] Bino RJ, Hall RD, Fiehn O, Kopka J, Saito K, Draper J, Nikolau BJ, Mendes P, Roessner-Tunali U, Beale MH, Trethewey RN, Lange BM, Wurtele ES, Sumner LW 2004 Potential of metabolomics as a functional genomics tool. Trends Plant Sci 9:418–425. doi:10.1016/j.tplants.2004.07.004. 15337491

[31] Jenkins H, Hardy N, Beckmann M, Draper J, Smith AR, Taylor J, Fiehn O, Goodacre R, Bino RJ, Hall R, Kopka J, Lane GA, Lange BM, Liu JR, Mendes P, Nikolau BJ, Oliver SG, Paton NW, Rhee S, Roessner-Tunali U, Saito K, Smedsgaard J, Sumner LW, Wang T, Walsh S, Wurtele ES, Kell DB 2004 A proposed framework for the description of plant metabolomics experiments and their results. Nat Biotechnol 22:1601–1606. doi:10.1038/nbt1041. 15583675

[32] Jenkins H, Johnson H, Kular B, Wang T, Hardy N 2005 Toward supportive data collection tools for plant metabolomics. Plant Physiol 138:67–77. doi:10.1104/pp.104.058875. 15888680 PMC1104162

[33] Quackenbush J 2004 Data standards for “omic” science. Nat Biotechnol 22:613–614. doi:10.1038/nbt0504-613. 15122299

[34] Fiehn O, Wohlgemuth G, Scholz M, Kind T, Lee DY, Lu Y, Moon S, Nikolau B 2008 Quality control for plant metabolomics: reporting MSI-compliant studies. Plant J 53:691–704. doi:10.1111/j.1365-313X.2007.03387.x. 18269577

[35] Lindon JC, Keun HC, Ebbels TM, Pearce JM, Holmes E, Nicholson JK 2005 The Consortium for Metabonomic Toxicology (COMET): aims, activities and achievements. Pharmacogenomics 6:691–699. doi:10.2217/14622416.6.7.691. 16207146

[36] Rubtsov DV, Griffin JL 2007 Time-domain Bayesian detection and estimation of noisy damped sinusoidal signals applied to NMR spectroscopy. J Magn Reson 188:367–379. doi:10.1016/j.jmr.2007.08.008. 17827043

[37] Donia MS, Cimermancic P, Schulze CJ, Wieland Brown LC, Martin J, Mitreva M, Clardy J, Linington RG, Fischbach MA 2014 A systematic analysis of biosynthetic gene clusters in the human microbiome reveals a common family of antibiotics. Cell 158:1402–1414. 25215495 10.1016/j.cell.2014.08.032 PMC4164201

[38] Moffitt MC, Neilan BA 2004 Characterization of the nodularin synthetase gene cluster and proposed theory of the evolution of cyanobacterial hepatotoxins. Appl Environ Microbiol 70:6353–6362. doi:10.1128/AEM.70.11.6353-6362.2004. 15528492 PMC525115

[39] Ziemert N, Lechner A, Wietz M, Millán-Aguiñaga N, Chavarria KL, Jensen PR 2014 Diversity and evolution of secondary metabolism in the marine actinomycete genus Salinispora. Proc Natl Acad Sci U S A 111:E1130–E1139. doi:10.1073/pnas.1324161111. 24616526 PMC3970525

[40] Harper DS, Loesche WJ 1983 Effect of pH upon sucrose and glucose catabolism by the various genogroups of Streptococcus mutans. J Dent Res 62:526–531. doi:10.1177/00220345830620050101. 6573364

[41] Kara D, Luppens SB, Cate JM 2006 Differences between single- and dual-species biofilms of Streptococcus mutans and Veillonella parvula in growth, acidogenicity and susceptibility to chlorhexidine. Eur J Oral Sci 114:58–63. doi:10.1111/j.1600-0722.2006.00262.x. 16460342

[42] Venable JD, Dong MQ, Wohlschlegel J, Dillin A, Yates JR 2004 Automated approach for quantitative analysis of complex peptide mixtures from tandem mass spectra. Nat Methods 1:39–45. doi:10.1038/nmeth705. 15782151

[43] Holden MT, Ram Chhabra S, de Nys R, Stead P, Bainton NJ, Hill PJ, Manefield M, Kumar N, Labatte M, England D, Rice S, Givskov M, Salmond GP, Stewart GS, Bycroft BW, Kjelleberg S, Williams P 1999 Quorum-sensing cross talk: isolation and chemical characterization of cyclic dipeptides from Pseudomonas aeruginosa and other Gram-negative bacteria. Mol Microbiol 33:1254–1266. doi:10.1046/j.1365-2958.1999.01577.x. 10510239

[44] Ortiz-Castro R, Díaz-Pérez C, Martínez-Trujillo M, del Río RE, Campos-García J, López-Bucio J 2011 Transkingdom signaling based on bacterial cyclodipeptides with auxin activity in plants. Proc Natl Acad Sci U S A 108:7253–7258. doi:10.1073/pnas.1006740108. 21482761 PMC3084137

[45] González JE, Keshavan ND 2006 Messing with bacterial quorum sensing. Microbiol Mol Biol Rev 70:859–875. doi:10.1128/MMBR.00002-06. 17158701 PMC1698510

[46] de Carvalho MP, Abraham WR 2012 Antimicrobial and biofilm inhibiting diketopiperazines. Curr Med Chem 19:3564–3577. doi:10.2174/092986712801323243. 22709011

[47] Chyan YJ, Poeggeler B, Omar RA, Chain DG, Frangione B, Ghiso J, Pappolla MA 1999 Potent neuroprotective properties against the Alzheimer beta-amyloid by an endogenous melatonin-related indole structure, indole-3-propionic acid. J Biol Chem 274:21937–21942. doi:10.1074/jbc.274.31.21937. 10419516

[48] Poeggeler B, Sambamurti K, Siedlak SL, Perry G, Smith MA, Pappolla MA 2010 A novel endogenous indole protects rodent mitochondria and extends rotifer lifespan. PLoS One 5:e10206. doi:10.1371/journal.pone.0010206. 20421998 PMC2858081

[49] Reiter RJ, Guerrero JM, Garcia JJ, Acuña-Castroviejo D 1998 Reactive oxygen intermediates, molecular damage, and aging. Relation to melatonin. Ann N Y Acad Sci 854:410–424. doi:10.1111/j.1749-6632.1998.tb09920.x. 9928448

[50] Li X, Xiong X, Yi C 2016 Epitranscriptome sequencing technologies: decoding RNA modifications. Nat Methods 14:23–31. doi:10.1038/nmeth.4110. 28032622

[51] McLean JS, Ona ON, Majors PD 2008 Correlated biofilm imaging, transport and metabolism measurements via combined nuclear magnetic resonance and confocal microscopy. ISME J 2:121–131. doi:10.1038/ismej.2007.107. 18253132 PMC4454505

[52] Dominissini D, Nachtergaele S, Moshitch-Moshkovitz S, Peer E, Kol N, Ben-Haim MS, Dai Q, Di Segni A, Salmon-Divon M, Clark WC, Zheng G, Pan T, Solomon O, Eyal E, Hershkovitz V, Han D, Doré LC, Amariglio N, Rechavi G, He C 2016 The dynamic N(1)-methyladenosine methylome in eukaryotic messenger RNA. Nature 530:441–446. doi:10.1038/nature16998. 26863196 PMC4842015

[53] Terleckyj B, Shockman GD 1975 Amino acid requirements of Streptococcus mutans and other oral streptococci. Infect Immun 11:656–664. 1091547 10.1128/iai.11.4.656-664.1975 PMC415118

[54] Tian Y, He X, Torralba M, Yooseph S, Nelson KE, Lux R, McLean JS, Yu G, Shi W 2010 Using DGGE profiling to develop a novel culture medium suitable for oral microbial communities. Mol Oral Microbiol 25:357–367. doi:10.1111/j.2041-1014.2010.00585.x. 20883224 PMC2951289

[55] Weisburg WG, Barns SM, Pelletier DA, Lane DJ 1991 16S ribosomal DNA amplification for phylogenetic study. J Bacteriol 173:697–703. doi:10.1128/jb.173.2.697-703.1991. 1987160 PMC207061

[56] Garg N, Kapono C, Lim YW, Koyama N, Vermeij MJ, Conrad D, Rohwer F, Dorrestein PC 2015 Mass spectral similarity for untargeted metabolomics data analysis of complex mixtures. Int J Mass Spectrom 377:719–717. doi:10.1016/j.ijms.2014.06.005. 25844058 PMC4379709

[57] Tan DX, Hardeland R, Manchester LC, Korkmaz A, Ma S, Rosales-Corral S, Reiter RJ 2012 Functional roles of melatonin in plants, and perspectives in nutritional and agricultural science. J Exp Bot 63:577–597. doi:10.1093/jxb/err256. 22016420

[58] Tilden AR, Becker MA, Amma LL, Arciniega J, McGaw AK 1997 Melatonin production in an aerobic photosynthetic bacterium: an evolutionarily early association with darkness. J Pineal Res 22:102–106. doi:10.1111/j.1600-079X.1997.tb00310.x. 9181522

[59] Jiao J, Ma Y, Chen S, Liu C, Song Y, Qin Y, Yuan C, Liu Y 2016 Melatonin-producing endophytic bacteria from grapevine roots promote the abiotic stress-induced production of endogenous melatonin in their hosts. Front Plant Sci 7:1387. doi:10.3389/fpls.2016.01387. 27708652 PMC5030213

[60] Wikoff WR, Anfora AT, Liu J, Schultz PG, Lesley SA, Peters EC, Siuzdak G 2009 Metabolomics analysis reveals large effects of gut microflora on mammalian blood metabolites. Proc Natl Acad Sci U S A 106:3698–3703. doi:10.1073/pnas.0812874106. 19234110 PMC2656143

[61] Zhang LS, Davies SS 2016 Microbial metabolism of dietary components to bioactive metabolites: opportunities for new therapeutic interventions. Genome Med 8:46. doi:10.1186/s13073-016-0296-x. 27102537 PMC4840492

[62] Ali L, Khan AL, Hussain J, Al-Harrasi A, Waqas M, Kang SM, Al-Rawahi A, Lee IJ 2016 Sorokiniol: a new enzymes inhibitory metabolite from fungal endophyte Bipolaris sorokiniana LK12. BMC Microbiol 16:103. doi:10.1186/s12866-016-0722-7. 27277006 PMC4899901

[63] Shannon P, Markiel A, Ozier O, Baliga NS, Wang JT, Ramage D, Amin N, Schwikowski B, Ideker T 2003 Cytoscape: a software environment for integrated models of biomolecular interaction networks. Genome Res 13:2498–2504. doi:10.1101/gr.1239303. 14597658 PMC403769

[64] Wishart DS 2016 Emerging applications of metabolomics in drug discovery and precision medicine. Nat Rev Drug Discov 45:473–484. doi:10.1038/nrd.2016.32. 26965202

[65] Consortium HMP. 2012 Structure, function and diversity of the healthy human microbiome. Nature 486:207–214. doi:10.1038/nature11234. 22699609 PMC3564958

[66] Consortium HMP. 2012 A framework for human microbiome research. Nature 486:215–221. doi:10.1038/nature11209. 22699610 PMC3377744

