# Supplementary material for: Metabolic Fingerprints from the Human Oral Microbiome Reveal a Vast Knowledge Gap of Secreted Small Peptidic Molecules
Source: mSystems. 2017 Jul 18;2(4):e00058-17. doi: 10.1128/mSystems.00058-17 (PMC5516222; doi:10.1128/mSystems.00058-17)
Supplement: FIG S1 [file sys004172116sf1.pdf]

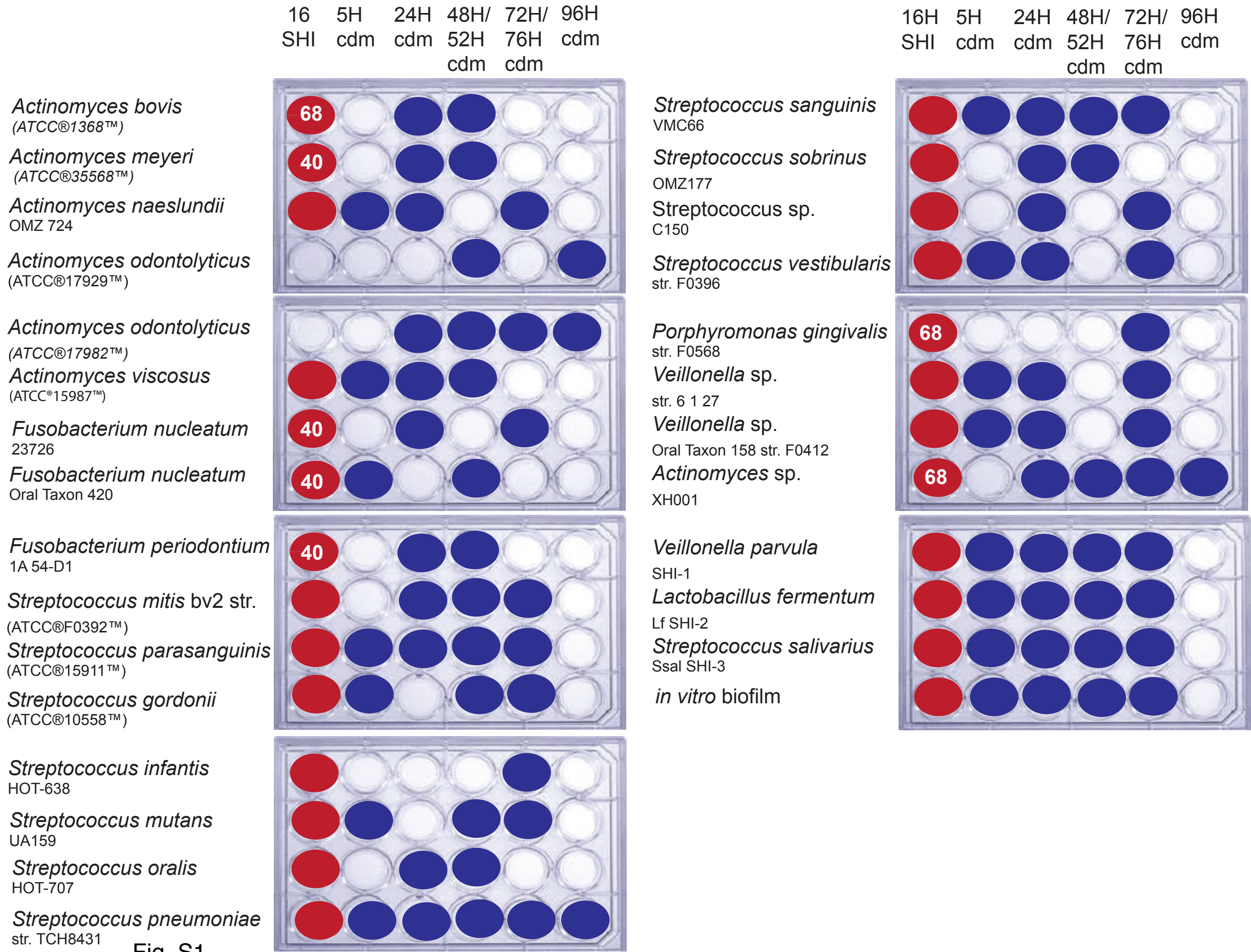
16 SHI
5H cdm
24H cdm
48H/ 52H cdm
72H/ 76H cdm
96H cdm
16H SHI
Actinomyces bovis (ATCC®1368™)
Actinomyces meyeri (ATCC®35568™)
Actinomyces naeslundii OMZ 724
Actinomyces odontolyticus (ATCC®17929™)
Actinomyces odontolyticus (ATCC®17982™)
Actinomyces viscosus (ATCC®15987™)
Fusobacterium nucleatum 23726
Fusobacterium nucleatum Oral Taxon 420
Fusobacterium periodontium 1A 54-D1
Streptococcus mitis bv2 str. (ATCC®F0392™)
Streptococcus parasanguinis (ATCC®15911™)
Streptococcus gordonii (ATCC®10558™)
Streptococcus infantis HOT-638
Streptococcus mutans UA159
Streptococcus oralis HOT-707
Streptococcus pneumoniae str. TCH8431
Streptococcus sanguinis VMC66
Streptococcus sobrinus OMZ177
Streptococcus sp. C150
Streptococcus vestibularis str. F0396
Porphyromonas gingivalis str. F0568
Veillonella sp. str. 6 1 27
Veillonella sp. Oral Taxon 158 str. F0412
Actinomyces sp. XH001
Veillonella parvula SHI-1
Lactobacillus fermentum Lf SHI-2
Streptococcus salivarius Ssal SHI-3
in vitro biofilm
68
40
40
40
40
68
68

Fig. S1
