# Supplementary figures and images for: Metabolic Fingerprints from the Human Oral Microbiome Reveal a Vast Knowledge Gap of Secreted Small Peptidic Molecules
Source: mSystems. 2017 Jul 18;2(4):e00058-17. doi: 10.1128/mSystems.00058-17 (PMC5516222; doi:10.1128/mSystems.00058-17)

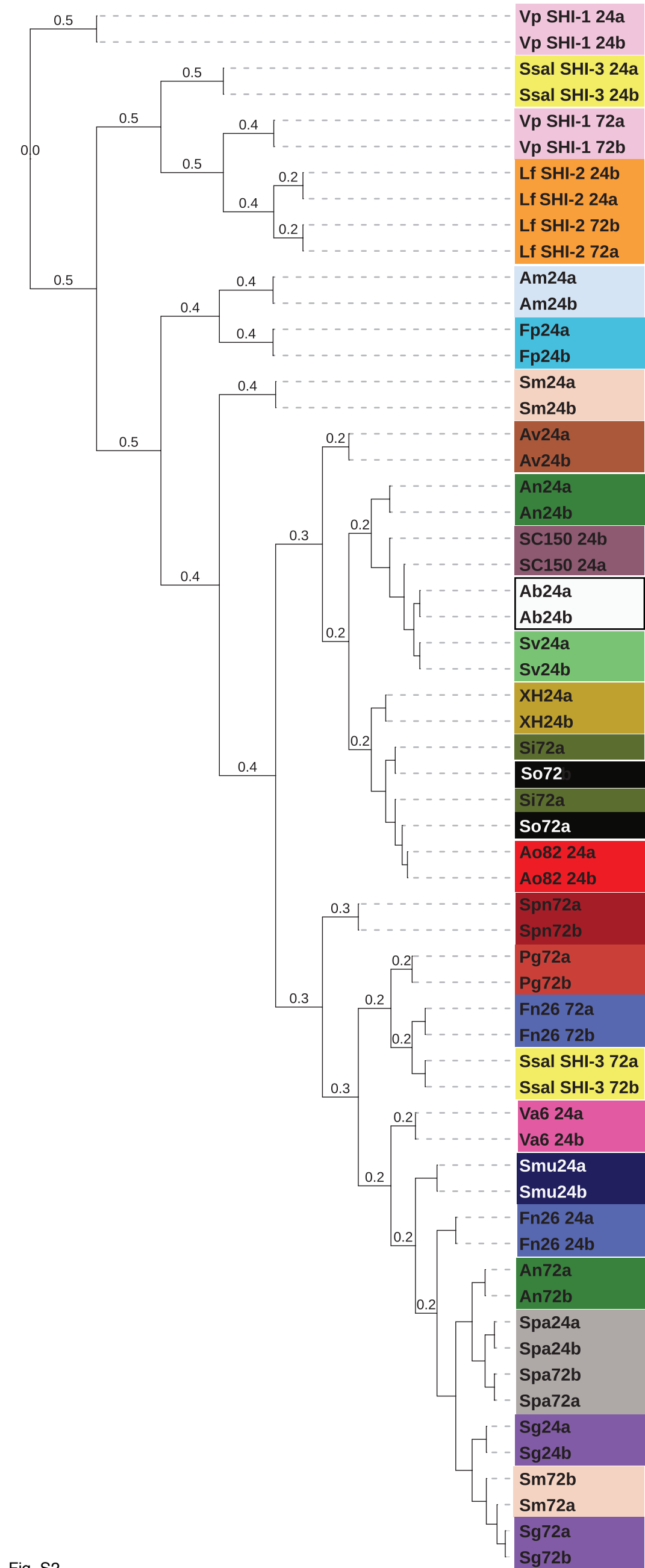

Fig. S2

Supplement: FIG S2 [file sys004172116sf2.pdf]

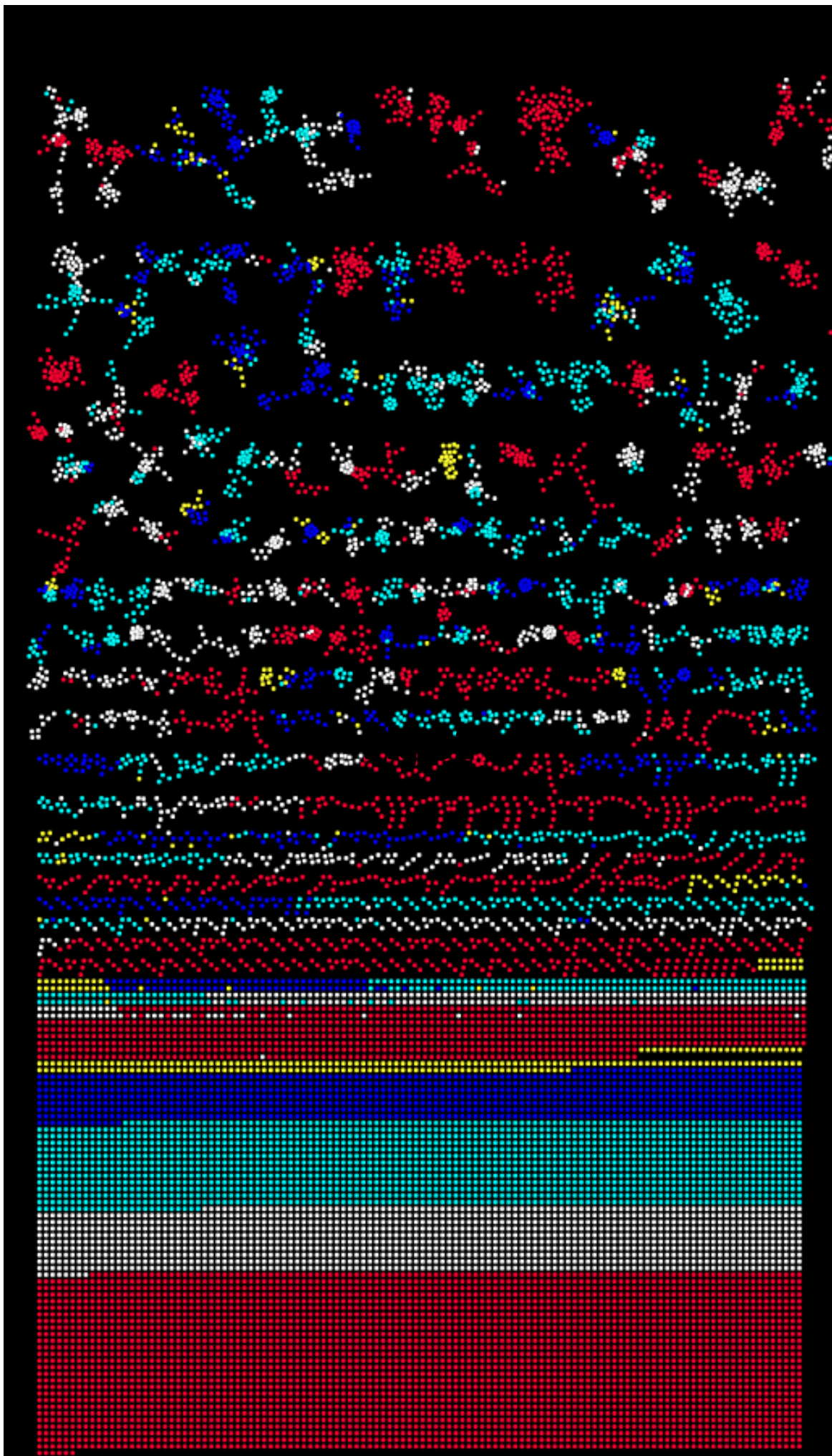

Fig. S3

Supplement: FIG S3 [file sys004172116sf3.pdf]
